# Factors influencing the help-seeking behavior in patients with mild cognitive impairment: a qualitative study

**DOI:** 10.1186/s12913-023-10281-5

**Published:** 2023-12-02

**Authors:** Yu-Chen Jiao, Jing Chang, Chang Liu, Shi-Yu Zhou, Yan Ji, Yao Meng

**Affiliations:** 1 School of Nursing, Nanjing Medical University, Nanjing, China; 2 Department of Radiotherapy, Jiangsu Cancer Hospital, Jiangsu Institute of Cancer Research, The Affiliated Cancer Hospital of Nanjing Medical University, Nanjing, China

**Keywords:** Help-seeking behavior, Influencing factors, Mild cognitive impairment, Qualitative study

## Abstract

**Background:**

The early diagnosis and intervention of mild cognitive impairment (MCI) patients is expected to delay the progression of AD. Delayed treatment will lead to MCI patients missing the best intervention expectation. At present, the medical help-seeking behavior of this group is not optimistic. This study aimed to explore influencing factors of help-seeking behavior among patients with MCI in China based on the help-seeking behavior model.

**Methods:**

Twenty-two patients with MCI were recruited to participate in semi-structured interviews via purposeful sampling with a qualitative, descriptive design. Data were analyzed by qualitative content analysis.

**Results:**

The study revealed the main influencing factors of help-seeking behavior among MCI patients in China included perceived disease threat, symptom attribution, disease knowledge, use of cognitive compensation strategies, sense of foreseeable burden, social support, economic condition, and accessibility of medical service.

**Conclusions:**

The help-seeking behavior of patients with MCI is affected by multiple factors. There are some key factors in different stages of the help-seeking process. Healthcare providers can utilize these factors to design targeted interventions for promoting early help-seeking of patients with MCI.

**Supplementary Information:**

The online version contains supplementary material available at 10.1186/s12913-023-10281-5.

## Background

With the acceleration in the global aging population, the prevalence and incidence of dementia are increasing at an alarming rate, seriously affecting healthy aging. According to the World Health Organization (WHO), the total number of patients with dementia worldwide will reach 152 million by 2050 [1]. Mild cognitive impairment (MCI), a stage of cognitive impairment between normal aging and dementia [2], is an optimal time to inhibit the progression of the disease. The prevalence of MCI is estimated to be 10–20% in older adults aged ≥ 65 years in the world [3]. In China, the prevalence of MCI in people over the age of 60 years old has reached 15.54% and is currently rising [4]. Studies found that seeking effective medical help at the MCI stage can control individuals’ cognitive decline, and delay the progression of dementia [5–7]. However, most people with MCI in China never sought help from healthcare professionals [4]. Therefore, understanding the factors that prompt patients with MCI to seek medical attention is crucial.

### Brief literature reviews

Help-seeking for health issues is generally characterized as problem-focused and planned efforts, including visiting healthcare specialists [8]. Previous studies have shown that people with MCI show poor help-seeking performance [9, 10]. A national, large-sample cross-sectional survey in China, showed that 6926 (97.2%) of the 7125 patients diagnosed with MCI never sought help from healthcare professionals [4]. Furthermore, other studies showed that it takes an average of 1.5–2.5 years from the time patients notice signs of cognitive impairment before they actually seek help, missing the optimal time for effective interventions [11–14]. Exploring the reasons behind the delay in seeking help among people with MCI is critical to promoting early help to address the issue.

Studies have developed models to analyze the influencing factors of help-seeking behavior. Andersen’s behavioral model of using health services suggests that help-seeking is affected by multiple factors, including environment, population characteristics, health behavior, and the outcome [15]. Allison, et al. collected feedback on the help-seeking intentions of 250 participants and noted that their assessment positively predicted help-seeking intention, indicative of their willingness to seek help [11, 16]. Begum, et al. investigated older adults’ help-seeking responses to memory complaints and identified their concerns and general perceptions of seeking help [17]. However, all these studies only consider how intention, beliefs, and other static factors affect an individual’s help-seeking behavior. These static models pose challenges in studying the factors influencing help-seeking behavior in patients with MCI. The process of help-seeking behavior is dynamic, encompassing the initiation of help-seeking intention to the actual implementation of seeking help [18]. In fact, it can take several months, or even up to a year, for individuals with MCI to transition from a desire to making the behavior of seeking assistance [16, 19]. However, with consistently reduced cognitive function and the significant reduction of rational cognitive components, it is challenging for individuals with MCI to maintain a fixed decision [20, 21]. In other words, as cognitive function declines, MCI patients’ perceptions and corresponding influencing factors of help-seeking behavior are constantly changing. Thus, it is necessary to subdivide the stages of help-seeking behavior and analyze the influencing factors in different stages in patients with MCI.

The help-seeking model proposed by Levkoff, et al. focuses on the psychology and behavioral change during the help-seeking process. It has been applied to analyze the help-seeking process of patients with Alzheimer’s disease (AD) and related disorders [22–25]. This model describes the process and is a comprehensive application of four behavioral phases: (1) disease and symptom experience, (2) symptom appraisal, (3) decision to seek care, and (4) contact with care providers [26]. This model may provide a theoretical basis for a further understanding of the help-seeking process of patients with MCI. Therefore, compared with other working models, we believe that this model is more suitable for describing the help-seeking process of patients based on previous extensive contact with patients with MCI.

### Aim of the study

This study investigated the influencing factors of MCI patients’ help-seeking behavior based on the help-seeking model [22], exploring the in-depth reasons behind the poor help-seeking performance of MCI patients. We believe that it can assist policymakers, researchers, and healthcare providers. A deeper understanding of the influencing factors in different stages of the help-seeking process will allow for more accurate development of supportive interventions and facilitate timely medical help-seeking for MCI patients.

## Methodology

### Design

This study used a qualitative and descriptive design based on the philosophical foundation of naturalistic inquiry [27], focusing on influencing factors of help-seeking behavior in MCI patients. We conducted semi-structured interviews with a sample of MCI patients, and then analyzed the data through qualitative content analysis. The study was approved by the Nanjing Medical University Ethics Committee (2021-No. 648). This study followed the Consolidated criteria for reporting qualitative research (COREQ) [28].

### Recruitment

From February to August 2022, we recruited patients with MCI who visited the Memory Clinic at the Neurology Department, affiliated with the Brain Hospital of Nanjing Medical University, Jiangsu Province, China, based on a purposive sampling approach. We sought potentially informative patients and documented the sample’s characteristics, including age, sex, and education level.

### Inclusion criteria

The criteria for inclusion into the study were: ***(a)*** having the symptoms of MCI [29], such as cognitive decline reported by the patient or informant or observed by an experienced clinician. Also, documented objective evidence of impairment from neuropsychological testing in one or more cognitive domains such as memory, language, executive function, visuospatial function, and independent activities of daily living function; but no dementia. ***(b)*** Primary school education level or above.

***(c)*** Good communication skills and hearing enough to go through the interview. In addition, we cooperated with neurological physicians and neuropsychological measurement researchers to jointly identify the patient eligibility criteria for MCI.

### Exclusion criteria

The criteria for exclusion were: ***(a)*** Patients with severe physical disease or serious mental disorder. ***(b)*** Patients who sought medical treatment for other diseases. ***(c)*** Patient’s refusal to be interviewed or to sign an informed consent.

### Study sample

A total of 22 patients with MCI were recruited for the purpose of this study, the characteristics of which are listed in Table 1. The sample included ten males and 12 females with MCI, and the ages ranged from 45 to 71 years old (average 59 years). Most patients had completed their secondary school education (N = 15). The self-reported memory decline of the study population ranged from two to 24 months (average, 10 months). The interview period ranged from 20 to 74 min (average, 34 min). The scores on the Mini-mental State Examination (MMSE) ranged from 20 to 30 (average, 26) [30]. The Montreal Cognitive Assessment Scale (MoCA) resulted in an average of 19 (range, 15–23) [31].

**Table 1:** Patients’ Demographic Characteristics

No.	Gender	Age	Education	Location	Self-reported memory decline time (months)	MMSE score	MOCA score
P1	Female	53	College	Urban	2	27	21
P2	Female	71	High school	Rural	12	26	21
P3	Female	69	College	Urban	24	27	18
P4	Female	63	Junior high school	Urban	6	30	17
P5	Female	66	High school	Urban	6	24	17
P6	Female	46	Junior high school	Urban	24	20	15
P7	Male	54	High school	Rural	24	27	21
P8	Female	61	Junior high school	Urban	3	28	15
P9	Male	58	College	Urban	6	26	18
P10	Male	61	College	Urban	3	26	20
P11	Male	45	High school	Urban	9	28	23
P12	Female	61	Primary school	Rural	24	26	20
P13	Male	50	High school	Urban	6	26	19
P14	Male	68	Junior high school	Urban	12	24	18
P15	Male	52	High school	Rural	6	26	21
P16	Female	60	College	Urban	10	28	16
P17	Female	56	High school	Urban	12	26	18
P18	Male	55	Junior high school	Urban	8	27	20
P19	Female	65	High school	Urban	18	24	17
P20	Male	62	High school	Urban	6	26	20
P21	Female	56	Highschool	Urban	3	25	16
P22	Male	60	College	Urban	6	24	18

*Key*: Mini-Mental State Examination: MMSE; Montreal Cognitive Assessment: MoCA

### Data collection

From March to September 2022, the first author completed data collection to ensure the homogeneity of interviews. All members of our research group had received the qualitative research courses, and mastered interview skills and data analysis methods. In addition, the corresponding author, a professor, had a relevant doctoral degree and rich qualitative research skills and experience to supervise the study team.

### Interview protocol

The interview protocol was developed according to the help-seeking model proposed by Levkoff, et al. [22]. The first author has conducted the formal interviews since February 2022, after a pilot interview (Supplementary material). The interview outline included four parts: ***(1)*** disease and symptom experience, ***(2)*** symptom appraisal, ***(3)*** decision to seek care, and ***(4)*** contacting the care providers. The corresponding author guided the interview process and modified the outline (Supplementary Material). Before the interview, we introduced the background and purpose of the study to patients and invited them to join our study based on voluntary, mutual trust and confidentiality principles. All of the patients confirmed their voluntary participation by reviewing and signing an informed consent. Then, patients were asked individually to complete a brief demographic survey. All interviews were conducted in an undisturbed and quiet office at the hospital.

### Interview language

The whole interview was conducted in Chinese language and recorded. We conducted interviews based on the established outline and appropriately responded, guided, and questioned to stimulate patients to express the influencing factors of help-seeking behavior clearly and comprehensively. At the same time, the researcher documented the patient’s significant views and non-verbal behaviors in the memo.

### Study team’s responsibilities

The first author was responsible for transcribing the audio tapes and integrating the memo notes within 24 h after the interviews. The other researchers checked the transcribed data, and provided the transcription text to patients, and verified them. Data collection and analyses were carried out simultaneously. When the qualitative data reached a thematic saturation level, we terminated the interview.

### Data analyses

We applied qualitative content analysis [33] to assess the interview data and managed all other data analyses with QSR Nvivo-12 software, version 30. The specific analytical steps of this study were as follows [32, 33]: ***1)****Preparation*: two researchers repeatedly listened to the interview audiotapes, read the transcribed data, and examined the original data to obtain a sense of integrity. ***2)****Organization*: we used inductive content analysis in this step. The researchers labeled the meaningful statements about influencing factors of the help-seeking behavior in patients with MCI line-by-line and then open-coded. Finally, we clustered and summarized the codes through similarities and differences and gradually formed the themes. ***3)****Reports*: the researchers re-read transcribed the data for simplification and collation. The final themes were determined after discussion and reporting the results to the study team members.

### Triangulation

We used triangulation in data analysis [34]. First, authors independently read, analyzed, and encoded each transcribed data, wrote reflection diaries at all times, and constantly reflected and compared the analysis results with the original data. In order to ensure the accuracy and credibility of the results and before reaching a consensus, the three researchers discussed the matters when any differences in coding and themes were identified.

## Results

**Fig. 1:**
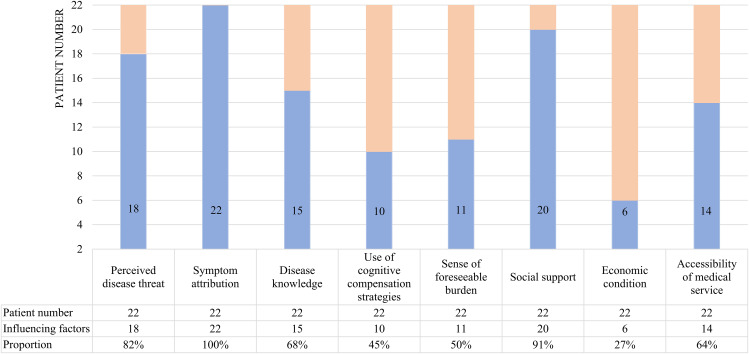
Influencing factors number of help-seeking behavior among 22 MCI patients

**Fig. 2:**
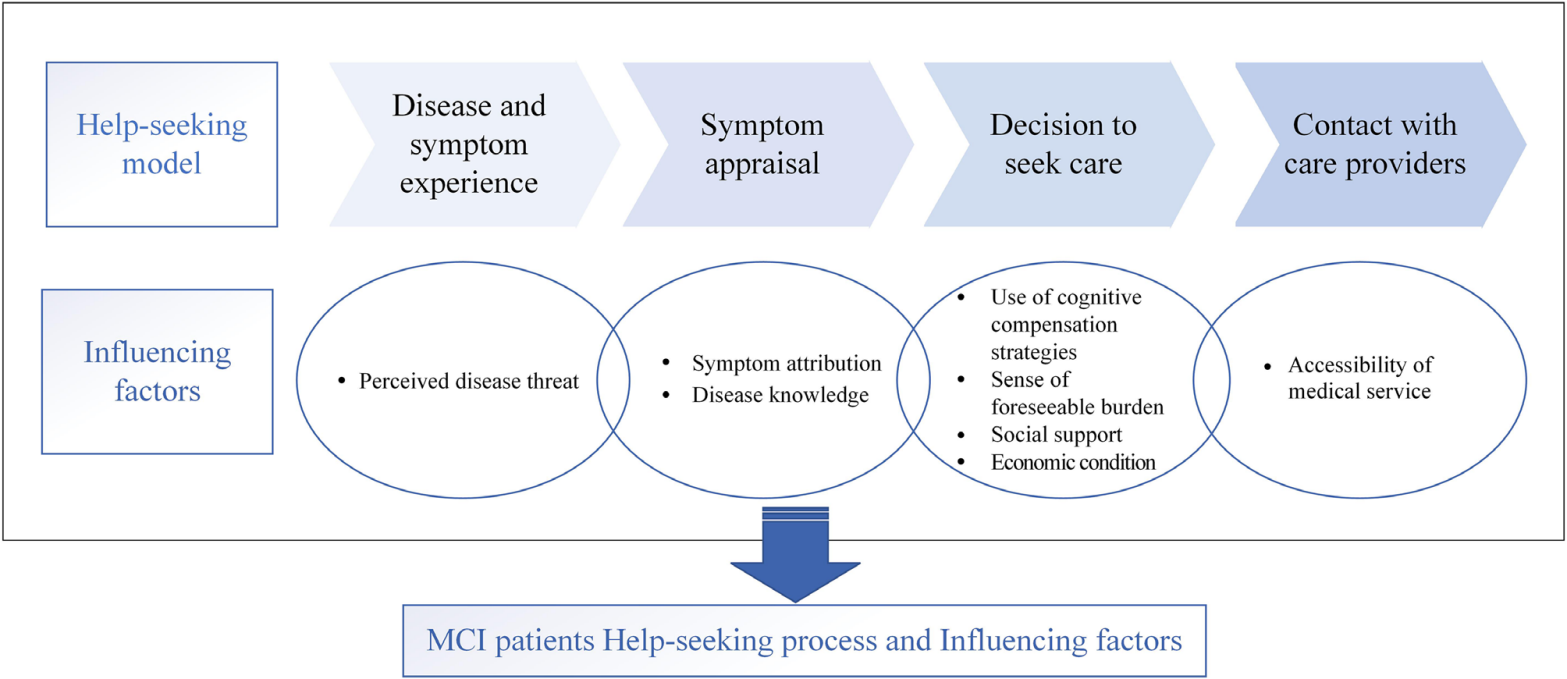
Factors influencing the help-seeking behavior among MCI patients

Through qualitative research, we found that eight influencing factors run through the four stages of the help-seeking process. They include perceived disease threat (18/22 patients, 82%), symptom attribution (22/22 patients, 100%), disease knowledge (15/22 patients, 68%), use of cognitive compensation strategies (10/22 patients, 45%), sense of foreseeable burden (11/22 patients, 50%), social support (20/22 patients, 91%), economic condition (6/22 patients, 27%), and accessibility of medical services (14/22 patients, 64%) (Fig. 1). At the same time, we extracted and reported each stage’s main factors based on the help-seeking behavior model(Fig. 2) [22].

### Perceived Disease threat

According to the results, 18 patients were more motivated to seek help if they believed the disease posed a threat to them. For example, P6: “When I got sick, I started having crazy thoughts and thinking in a bad direction. I think I should go to the hospital. If anything happens, I can know it earlier.“ However, individuals who perceive a threat of disease increased their desire to seek help under two key conditions. For one thing, their perceived threats were accompanied by negative emotions such as anxiety, fear of dementia, and so on. P1: “I am anxious right now. I think I will have dementia in the future, and I cannot expect my husband to take care of me…”. P3: “I felt scared and worried about developing dementia because it would make me have to obey the command of others and cannot do anything by myself…”. For another, patients who recognized that their memory problems stemmed from cognitive decline were more inclined to proactively pursue help-seeking. P7: “I went shopping and found that I did not bring the key downstairs. It is not once or twice; this happens more and more often. It is causing me much trouble so I feel abnormal. “P11: “I was admitted to the hospital because of poor memory and lack of concentration. I made three mistakes in a month during work. I have worked for decades, and I did not make a mistake! Now this has attracted my attention and I wanted to see the doctor.“ In addition, the results showed that this perceived disease threat primarily occurs during the initial stage of the help-seeking process, known as the stage of disease and symptom experience. As a result, perceived disease threat emerges as a significant influencing factor in the help-seeking process, particularly during *the stage of disease and symptom experience*.

### Symptom attribution

Symptom attribution refers to the patients’ self-recognition of the cause of their symptoms. Our study found that patients with MCI actively seek help under two kinds of attribution. First, some patients attributed their memory decline to cognitive disorders. P1: “I’m losing track of things. Maybe my brain function is getting broken.“ Second, someone was curious about the cause of their symptoms even if they were unaware of it. P9: “I felt something wrong with my brain and memory and wanted to see the doctor.“ P11: “We want to find the cause of the disease and treat it as soon as possible.“ However, patients with MCI did not actively seek help based on another two kinds of attribution. In the first place, if they rationalized their symptoms and viewed memory loss as a normal part of aging, they were less inclined to seek assistance and more likely to be encouraged by their family to consult a doctor. P2: “I think it is normal to have a poor memory because I am old.“ P16: “There is nothing wrong with me. It is not as serious as others say. Compared with young people, I am old and have a normal memory.“ P14: “I do forget things, but it’s not serious; I’m old and it’s okay. In the second place, patients who attributed their symptoms to recent stressful life events were also less inclined to actively seek help for their cognitive problems. P21: “I haven’t been sleeping well recently. My mother passed away a while ago, and I am very sad. This incident has stimulated me. I feel like I can’t remember things… " P5: " Recently, my upstairs’ neighbors quarreled at night. They are very noisy. Because of this, I have a headache along with poor memory. But I am optimistic and believe it’ll get better after a while; it won’t get worse”. In addition, results indicated that symptom attribution primarily occurs during the second stage of the help-seeking process, known as *the stage of symptom appraisal*. Therefore, distinctive attributions of symptoms influence the behavior of help-seeking.

### Disease knowledge

Patients with MCI are better able to identify symptoms and seek assistance when they have knowledge of dementia. P1: “I think I have bad symptoms, which will develop into dementia in the future.“ P3: “I read some books and articles about the disease. My symptoms are similar to pre-dementia. I am worried that if the disease continues to develop, I may get dementia (waving hands and shaking heads)”. However, patients who lacked knowledge about dementia were less likely to seek help, and they had difficulty recognizing disease symptoms or thought help-seeking was unnecessary. P12: “We basically did not go to school, were poorly educated, and didn’t understand, so it took us a long delay.“ P5: “I don’t understand the disease. My husband brought me here. What’s wrong with me?“ P17: “My daughter said I might have dementia. Sometimes she asks me where things are, I can’t remember, but I think it’s just joking. I don’t think there’s any problem with me… We just know there is a disease called Alzheimer’s disease. We don’t know what it is, clearly.“ P6: “I feel that my memory is not very good, but I don’t think it’s a big deal, you know, the symptom was too small to see a doctor.“ Meanwhile, the importance of disease knowledge is also more reflected in *the stage of symptom appraisal*. Therefore, disease knowledge also influences patients’ help-seeking behavior.

### Use of cognitive compensation strategies

Cognitive Compensation Strategies refer to taking actions to remember specific things [35]. Our study showed that using cognitive compensation strategies frequently could be a major obstacle to seeking medical help. Interestingly, some patients may seek medical attention for other reasons (e.g., perceived disease threat), but they tend to reduce their desire for help when they rely on cognitive compensation strategies. P7: " I think I was having memory problems, but I have methods to cope with it. Sometimes when my family needed more food, I only remembered four kinds of vegetables, as if five kinds of things were a barrier for me. Luckily, I can use my mobile phone to remind me when I want to buy something.“ P11: “I am a driver, and I worked with an escort. When I forgot the way, he could remind me and show me the right direction, so the memory decline did not affect me too much.“ Therefore, the use of cognitive compensation strategies might inhibit help-seeking behavior in patients with MCI.

### Sense of foreseeable burden

Results showed that the sense of foreseeable burden might increase help-seeking behavior in patients with MCI. Sense of foreseeable burden refers to negative psychological feelings caused by individuals’ concern about how their illness and care needs may impact others [36]. The fear and guilt of being a burden to their offspring, and the fear that their young children would be left unattended urged the patients to make medical decisions for help-seeking. P3: “My son is filial and he often told me that I don’t need to worry about anything and he would do it for me. However, my son should focus on his career. Sometimes, I cannot sleep and think if my illness drags down my offspring and what should I do if I am paralyzed in the future?“ P4: “The disease is too painful for my family and me. I do not care, but it become a trouble now because it is a burden on my offspring. Now, the most important thing for me is to deal with this.“ Patients may feel distressed by the sense of burden, but it may also encourage them to seek help.

### Social support

On one hand, reminders from relatives and friends can motivate patients to engage in medical help. P8: “I’m living with my daughter. My son-in-law picks me up from the hospital every night. All my family members told me that I was sick and should go to hospital. " P3: “I told them (friends in the community) about my disease because we have a good relationship. They also said that I should pay attention to the disease and get medical help…” P2: “My family insisted on me coming…” P5: “My husband thought that I had memory loss. He went straight to the hospital, got the admission certificate, and tricked me into going here. " On the other hand, our study found that emotional support from others could effectively facilitate patients’ medical help-seeking. P1: “My husband is very supportive of me going to the hospital. He said if you were all right, we shall rest easy.“ P12: “My daughter and son also found something wrong (disease symptoms) with me. Both urged me to go to the hospital and said they feared I would be lost in the future (Putting a hand over her mouth and laughing). " However, the indifferent or ridiculing attitude to the memory decline among people around the patients can influence their own attitude and hinder their willingness to seek medical treatment. P7: “At first, I did not take the disease symptoms seriously, because my peers and sisters had the symptoms, and they said they all had the symptoms, and that was normal, and I should not be nervous about it. They comforted me all the time.“ P4: " Speaking with friends about it prior to seeing the doctor gave me the impression that it is a shame thing to visit the hospital.“ Therefore, positive social support promotes the patients’ help-seeking behavior, whereas negative social support does not.

### Economic condition

According to our results, patients with MCI who had stable sources of income, better economic circumstances, and urban employee medical insurance (higher reimbursement ratio) are more likely to decide to seek assistance and display the corresponding behaviors. P12: “My daughter works for Huawei Company and has a good salary. She told me that I had to be cured so that I could have a future life. I will take care of my grandchildren in the future.“ P22: “I am living in Nanjing and my home is close to the hospital. I’d like to have a full body exam because my health insurance will cover the cost. I don’t have any economic pressures…” As a result, the economic condition is also one of the influencing factors of help-seeking behavior.

### Accessibility of medical services

Accessibility of Medical Services reflects the medical resource distribution [37]. Our study found that patients who had easy access to medical resources tended to have a better willingness to go to the hospital. P19: “I’m a native. It’s convenient for me to see the doctor.“ P4: " I came to the hospital this time because there were some abnormalities in the results of my previous CT scan. By the way, I came to verify my memory ability because I was a little scared.“ In addition, cognitive testing is not a common screening program among Chinese residents, and primary medical services specifically targeting MCI have not been effectively implemented in rural areas in China. P7: “Yes, there is a policy in place for annual physical examinations, but it mainly focuses on basic measurements such as height, weight, and blood pressure. Other tests are not typically conducted during these examinations.“ P15: " If a nurse or doctor regularly visited us, providing guidance and support, and if the fees for their services were fair and reasonable, or possibly reimbursable for certain expenses, we would certainly be willing to cooperate.“ As a result, the accessibility of medical services also affected behaviors of help-seeking among patients with MCI. In addition, the results showed that this accessibility of medical services primarily occurs during the last stage of the help-seeking process, known as *Contact with Care Providers*.

## Discussion

This study explored the help-seeking process in patients with MCI and identified the factors that would impact the help-seeking behavior based on the qualitative research data [22]. Eight critical factors were involved in the help-seeking process, including *perceived disease threat, symptom attribution, disease knowledge, use of cognitive compensation strategies, sense of foreseeable burden, social support, economic condition, and accessibility of medical services*. We also extracted the main influencing factors at each stage of the help-seeking process. Based on our convincing evidence, we believe that it is imperative to use our proposed guideline as a starting point to encourage patients with MCI to seek medical attention.

### Perceived Disease threat

According to our results, patients with MCI were more inclined to seek assistance if they believed that the disease threat was significant. Consistent with our findings, Mechanic, et al. proposed a general theory of help-seeking behavior and identified ten key determinants of help-seeking behavior, one of which is the perceived risk of symptoms [38]. Further, we discovered that when patients with MCI perceived threats along with accompanying emotional symptoms, they were more likely to prompt them to engage in help-seeking behaviors. The perceived threat and accompanying emotional disturbances prompt them to decide to seek medical help, consistent with the findings suggested by Gigi A, et al. [39]. While negative emotions may encourage helpful behaviors, we also expect that medical professionals will attend to their patients’ unpleasant feelings and uphold their mental health. Meanwhile, the results also showed that a clear understanding and awareness of the disease is an essential influencing factor that prompts them to seek help. This finding further confirms that individuals with MCI often retain relatively intact cognitive abilities, making it an opportune time for early intervention against the illness.

### Symptom attribution

Our results showed that patients with MCI will actively seek help when they recognize their own cognitive decline and express a desire to understand the underlying cause. However, many patients did not actively seek help because they thought their memory loss was a natural part of aging or the result of stressful situations that happened suddenly. In fact, when people have memory issues, they tend to assume what the symptoms mean and try to interpret them as they want [26]. It is true that Chinese people traditionally view dementia as a part of normal aging and often do not believe they should seek medical help [40]. A study showed that dementia patients relate the symptoms to non-physiological causes, including psycho-social, paranormal phenomena, and normal aging [22]. Meanwhile, patients who attributed their symptoms to recent stressful events were also less likely to actively seek help. Despite the evidence that negative emotions caused by stress can have an impact on cognitive function [41], we believe that seeking help is still the fastest and most effective way to relieve symptoms. Thus, currently, there is a need to enhance people’s awareness and judgment of the symptoms of cognitive impairment.

### Disease knowledge

Our interviews also confirmed that sufficient knowledge about dementia is one of the reasons to encourage patients to seek help. A previous study showed that patients with poor health knowledge often have difficulty detecting the symptoms or they have an indifferent attitude toward them, thus limiting their help-seeking behavior [42]. Our research indicated that individuals with MCI who lack sufficient knowledge about the disease were more likely to deny or avoid acknowledging their symptoms, leading to their disease bias. Public health professionals should take steps to enhance educational interventions for memory problems and address cognitive misunderstandings and biases among individuals at risk for or already experiencing memory problems. For instance, to distinguish between pathological memory loss and typical aging, community nurses in China might frequently host health information seminars.

### Use of cognitive compensation strategies

Our results indicated that using cognitive compensation strategies might inhibit help-seeking behavior in patients with MCI. As part of therapeutic interventions, the use of cognitive compensation strategies typically consists of three approaches: *internal help* (e.g., psychological imagery), *external help* (e.g., using a list of items or calendars), and *reliance on assistance* (e.g., receiving reminders from others) [36]. All these strategies have been widely used in interventional studies on patients with MCI and they can address memory loss so that patients do not feel the influence of cognitive decline on their daily activities [43]. However, our current study found that using compensation strategies may prevent patients from seeking medical help. Prior research noted that the strategies discourage patients from seeking medical help by lowering their self-motivation [35]. Therefore, healthcare workers play a crucial role in helping patients recognize that the unconscious and frequent utilization of cognitive compensation strategies to cope with memory decline may be a warning sign of an approaching dementia.

### Sense of foreseeable burden

The sense of foreseeable burden might increase help-seeking behavior in patients with MCI. In fact, the sense of foreseeable burden, i.e., the prescient emotional burden, comes from the concept of the self-perceived burden and frequently occurs between patients and their family [44]. We found that the Chinese culture of filial piety (Children must support their parents when they are elderly) was the primary cause of this burden among patients with MCI. This makes parents fearful that when they are sick, they will be a burden to their children [45]. Hence, the sense of foreseeable burden prompts patients to seek medical care early. This finding has received limited attention in previous studies, possibly because they primarily focused on external factors such as patient characteristics, family social support, and access to medical resources and services. As a result, the emotional bond between patients and their relatives was not thoroughly explored [46].

### Social support

The results revealed that positive social support promoted the patients’ help-seeking behavior, whereas negative support did not. Self-regulation theory states that internal and external factors influence disease management [47]. As a part of the management, help-seeking behavior is also influenced by patients’ individual and social factors. We found that positive social support, especially family emotional support, promoted patients’ access to health care. Consistent with our results, adequate social support can help patients make early decisions about health care [48]. Studies have also shown that after perceiving memory symptoms, patients prefer seeking help from their spouses and children first rather than directly seeking medical help [49]. Family caregiver support may play a greater positive role than other support systems in the help-seeking process of patients [50]. It may be due to the traditional concept of “*home*” in China.

Chinese tradition believes that individuals are not entirely independent but belong to the family and strongly emotionally depend on family members. Khakbazan, et al. indicate that whether patients seek medical help after discovering symptoms depends on the evaluation of symptoms by their family members or friends, which is consistent with our study findings [51]. Thus, we propose that healthcare providers pay ample attention to the supportive needs of families with patients suffering from MCI. This can be achieved by enhancing emotional support between patients and their family members.

### Economic condition

The economic condition is also a crucial factor that influences the help-seeking behavior in patients with MCI. They only visit the hospital when their sickness worsens due to the high cost of examination and treatment. Patients with poor economic status were more likely to delay seeking medical help, which was consistent with previous research [52]. In addition, low-income patients often must work longer hours or at a higher intensity, which can make them less attentive to their health conditions and needs.

### Accessibility of medical services

Our study found that when access to medical services is limited, patients are less likely to seek help. Even if the patients had made a help-seeking decision, there were still influencing factors in the later stages of the help-seeking behavior model [22], i.e., contacting care providers. So, this is the reason why patients spoke more topic about access to medical services during the last stage of the help-seeking process. In China, there is an imbalance in the allocation of high-quality medical resources, with many of these resources being concentrated in tertiary hospitals. As a result, primary hospitals often lack the necessary resources to provide strong medical services [53]. This situation leads to difficulty in seeing a doctor for patients with MCI who live in remote areas [54]. Besides, medical staff in primary hospitals lack the sensitivity to cognitive decline, thus creating a vicious cycle.

All in all, according to our interview results, Firstly, it is recommended that Chinese health officials support the establishment of varying degrees of medical facilities in different regions, such as memory clinics. This is conducive to reducing medical costs and burden on families and society. Secondly, implementing medical treatment through internet platforms, remote consultations, shared patient databases, and establishing a two-way referral process can facilitate collaborative patient management among tertiary, secondary, and primary hospitals [55]. Thirdly, caregivers and social workers should play a role in propagandizing knowledge about cognitive impairment. Lastly, enhancing the training of medical practitioners in grassroots community healthcare institutions and raising their sensitivity towards MCI can significantly contribute to the early diagnosis of patients with MCI.

## Conclusions

This study revealed the main influencing factors of the help-seeking behavior in Chinese patients with MCI. The main factors included perceived disease threat, symptom attribution, disease knowledge, use of cognitive compensation strategies, sense of foreseeable burden, social support, economic condition, and accessibility of medical services. All the aforementioned factors can influence patients’ help-seeking behaviors, and the significance of each factor may vary at different stages of the help-seeking process. It is essential for experts to carry out interventions on the major determinants that encourage patients with MCI to seek assistance.

## Limitations

This study had some limitations. First, we only recruited MCI patients from urban areas in China, which restricted the sample representation and results. Therefore, more samples from rural areas and small towns would be desirable in the future. In addition, all interview data in our study were from the patient’s memory and dictation, so there might be some memory bias. Finally, the factors influencing the help-seeking behavior of MCI patients may not be comprehensive in our study. We will include non-help-seekers of MCI patients to research the help-seeking behavior of MCI patients and conduct behavioral motivation analysis in the future. Despite the above limitations, this study conducted qualitative research to comprehensively explore the main factors influencing the process of MCI patients’ medical help-seeking in China, based on the help-seeking behavior model. The findings is likely to enrich the existing domestic and foreign research results and laying a foundation for the subsequent promotion of MCI patients seeking professional medical help as early as possible to prevent the decline in cognitive function.

### Electronic supplementary material

Below is the link to the electronic supplementary material.

Supplementary Material 1

## Data Availability

Data are available upon reasonable request to the corresponding author (yanji@njmu.edu.cn), with Research Ethics Board approval.

## Abbreviations

MCI: Mild cognitive impairment
MMSE: Mini-mental State Examination
MoCA: Montreal Cognitive Assessment Scale
SRQR: Standards for Reporting Qualitative Research
