# Supplementary material for: Factors influencing the help-seeking behavior in patients with mild cognitive impairment: a qualitative study
Source: BMC Health Serv Res. 2023 Dec 2;23:1345. doi: 10.1186/s12913-023-10281-5 (PMC10693691; doi:10.1186/s12913-023-10281-5)
Supplement: Supplementary file 1 — Supplementary Material 1 [file 12913_2023_10281_MOESM1_ESM.docx]

**Supplementary Material**

**The influencing factors of help-seeking behavior in patients with mild cognitive impairment in China: a qualitative study**

**S1: Qualitative Interview Outline**

| **Phase 1：Disease and symptom experience** |
| --- |
| **a.** How did you firstly discover your condition? |
| **b.** What are your expectations, thoughts, perceptions and feelings about this disease or symptom?  **c.** How do other family members or others around you perceive the illness or symptoms? What are your thoughts and feelings?  **d.** Did you make any psychological or behavioral adjustments or responses before deciding to seek medical attention? |
| **Phase 2：Symptom appraisal** |
| **a.** When and why did you start asking for help? |
| **b.** What do you think is the main reason and motivation for you to ask for help?  **c.** What do you and your family know about cognitive therapy? Are cognitive health services available in your community? |
| **Phase 3：Decision to seek care** |
| **a.** What did you think after you made the decision to ask for help? What aspects are taken into consideration?  **b.** How do you feel about coming to the hospital and what are your expectations for treatment?  What factors do you think may hinder or promote the behavior of seeking medical help? |
| **Phase 4：Contact with care providers** |
| **a.** What did you tell the doctor about your symptoms? How do you feel and think when you communicate with the doctor?  **b.** What needs, expectations, or questions do you have for follow-up help and treatment? |
